# A prospective study of the effect of antihypertensive medications on the sexual functions of hypertensive adult male patients

**DOI:** 10.2144/fsoa-2020-0030

**Published:** 2020-06-02

**Authors:** Akinwumi Akinyinka Akinyede, Obiyo Nwaiwu, Olumuyiwa John Fasipe, Adedunni Olusanya, Sunday Olufemi Olayemi, Bisola Akande

**Affiliations:** 1 Department of Pharmacology, Therapeutics & Toxicology, Faculty of Basic Medical Sciences, University of Lagos, Idi-Araba, Lagos State, Nigeria; 2 Department of Clinical Pharmacology & Therapeutics, Faculty of Basic Clinical Sciences, University of Medical Sciences, Ondo City, Ondo State, Nigeria

**Keywords:** angiotensin-converting enzyme inhibitors, antihypertensive medication use, calcium channel blockers, diuretics, hypertensive adult male patients, sexual functions

## Abstract

**Background:**

The potential for antihypertensive medications to produce deleterious adverse effects on sexual functions among hypertensive adult male patients has been widely reported, such adverse effects may limit drug adherence and compliance.

**Aim:**

The aim of this study was to assess the effect of antihypertensive medication use on sexual functions among hypertensive adult male patients.

**Methodology:**

The study was carried out at the outpatient clinic of a Nigerian University Teaching Hospital. A total of one hundred and fifty-nine recruited hypertensive adult male patients that were being managed at the center over a 3-month period between January 2017 and April 2017 participated in the study; provided they satisfied the inclusion and exclusion criteria for enrolment.

**Results:**

The respondents were between 30 and 98 years of age, (mean of 59 ± 11.1 years). Blood pressure recorded was during their initial medical diagnosis for hypertension. Systolic blood pressure recorded was between 128 and 194 mmHg (mean of 162 ± 16.4 mmHg), while their diastolic blood pressure was between 78 and 120 mmHg (mean of 95 ± 10.7 mmHg). The highest occurrence of sexual dysfunctions was associated with calcium-channel blockers in 32 (20.1%) patients, followed by diuretics in 27 (17.0%) and, angiotensin-converting enzyme inhibitors in 20 (12.6%) patients.

**Conclusion:**

Calcium channel blockers caused the highest occurrence of sexual dysfunctions.

## Lay abstract

This prospective study assessed the effect of antihypertensive medications on sexual functions among hypertensive adult male patients attending the medical outpatient clinic of a Nigerian University Teaching Hospital. A total of 159 patients who consented to the study and had been attending the clinic regularly for at least 6-month were enrolled. The respondents aged 30–98 years (mean 59 ± 11.1 years). Antihypertensive medication-associated sexual dysfunctions among the patients were observed in descending order as follows: calcium channel blockers in 32 (20.1%), diuretics in 27 (17.0%), Angiotensin converting enzyme inhibitors in 20 (12.6%), centrally acting antihypertensives in 11 (6.9%), beta receptor blockers in 8 (5.0%), vasodilators in 2 (1.3%), combined alpha- and beta-receptor blockers in 2 (1.3%) and alpha receptor blockers in 1 (0.6%). Antihypertensive medical prescriptions should reflect a balance between efficacy and tendency to cause sexual dysfunctions.

Hypertension results from increased peripheral vascular arteriole resistance, increased smooth muscle tone and reduced capacitance of the venous system [1–3]. Most evidence implicates either disturbance in the kidneys' salt and water regulatory mechanisms (particularly abnormalities in the intrarenal renin–angiotensin system) and/or abnormalities of the sympathetic nervous system. These mechanisms are not mutually exclusive, and it is likely that both contribute, to some extent, in most cases of essential hypertension [4–6]. It has also been suggested that chronic endothelial dysfunction (injury) and vascular inflammation may also contribute to increased peripheral vascular resistance, increased vascular tone and vascular damage in hypertension as a result of the aging process, coupled with arteriosclerosis of the blood vessel walls [7–9].

Hypertension affects a significant number of the Nigerian population and many of these affected individuals have no symptoms [10,11]. Poorly controlled chronic hypertension (either systolic or diastolic) can lead to sexual dysfunctions [12], stroke [13], congestive heart failure [14], myocardial infarction [15] and renal failure [16]. A person is said to be hypertensive when there is a persistent and sustained increase in the resting blood pressure above or equal to (≥) 140/90 mmHg [17,18]. Hypertension is considered to be one of the most prevalent diseases in elderly patients (usually 60 years and above) and has been reported to affect 8–46.4% of the Nigerian population, according to the *World Journal of Cardiology* [19–21]. According to the World Health Statistics in 2009, the prevalence rates are quite similar between both men and women, varying between the range of 7.9–10.2% for men and 3.5–6.8% for women [22–24]. As hypertensive patients are more likely to have multiple comorbid conditions and are required to maintain stringent control of blood pressure, elderly Nigerian hypertensive patients are more likely to use multiple medications with attendant risk of polypharmacy [25–28].

It is recognized that some adverse drug reactions are restricted to particular drugs, while some are generalized and nonspecific adverse drug reactions. Based on the hypothesis that the administration of relatively low doses of multiple medications results in a higher tolerability than administration of relatively high doses of one medication, current clinical guidelines for the treatment of hypertension recommend combination therapies if needed, thus increasing the potential for polypharmacy in hypertensive patients [29–33]. The management of hypertensive patients should consider the impact of antihypertensive drugs on patients' sexual functions, the deterioration of which may result in reduced adherence to treatment [34–36]. Quality of life is recognized as a multifactorial variable, which encompasses adequate sexual functions as part of its components; it is important to pay attention to the symptomatic wellbeing, activity and sexual functions of these hypertensive adult male patients [37–40].

The results of some large trials that specifically evaluated the long-term effects of antihypertensive medications on sexual functions in older people demonstrate that the question about whether some classes of antihypertensive agents are more beneficial or harmful than the others remain largely unanswered and controversial [41–43]. Results from the long-term use of diuretics have been associated with adverse effects on sexual functions. Furthermore, thiazide diuretics and other diuretics have been often reported to be associated with sexual dysfunctions in men, including decreased libido, erectile dysfunction, intercourse dissatisfaction, orgasmic dysfunction and difficult ejaculation, when compared with other antihypertensive drug classes [44–46]. In addition, thiazide diuretics had been associated with impaired glucose metabolism and dyslipidemia [47,48]. Nonselective lipophilic beta adrenoreceptor antagonists, such as propanolol have been reported to produce negative effects that include sexual dysfunctions, depression and impairment of memory functions [49,50]. Angiotensin converting enzyme (ACE) inhibitors have been reported to exert favorable effects on the protection of end-organs but produce adverse effects including a dry unproductive cough, angioneurotic edema, hypotension – especially first dose related hypotension and congenital abnormality in some cases [51,52]. These drugs seem to be effective in maintaining or even improving cognitive function through mechanisms other than blood pressure control. In addition, a number of studies reported favorable impact of ACE inhibitors on sexual functions [53,54]. Currently, no class of antihypertensive medications clearly produces superior effects over the others in terms of preservation of sexual functions [50,51,53,54].

This study was designed to assess the effect of prescribed antihypertensive medications on the preservation of adequate sexual functions among hypertensive adult male patients attending the medical outpatient clinic of Lagos University Teaching Hospital, (LUTH), a tertiary healthcare facility in Nigeria.

## Methods

This was a descriptive, prospective study carried out at the medical outpatient clinic of LUTH, Lagos state Nigeria. This hospital receives referrals from within and outside the state. The recruited participants were known hypertensive adult male patients, who had been on antihypertensive medications for at least 6-month duration prior to their enrolment in this study. In addition, we also ensured that all the participants had adequately controlled blood pressure of <140/90 mmHg to properly eliminate the effect of poorly controlled chronic hypertension (either systolic or diastolic), which can lead to hypertension-induced sexual dysfunctions. The reason for enrolling patients taking antihypertensive medications for at least 6-month duration in this study was to allow time for the occurrence/development of the adverse effect(s) of antihypertensive medication-induced sexual dysfunctions among the respondents prior to their recruitment contacts. While at the same time, the respondents' blood pressure would have been adequately controlled to less than <140/90 mmHg. This will further ensure that the sexual dysfunctions observed in the enrolled participants were actually due to antihypertensive medication-induced sexual dysfunctions, and not from other pathological causes.

A total of 159 hypertensive adult male medical patients were recruited for the study and were being managed at the center over a 3-month period between January 2017 and April 2017. While hypertensive adult male medical patients who declined from participating in the study, hypertensive adult male medical patients with cognitive impairment, patients below the age of 18 years, newly diagnosed hypertensive adult male medical patients that were yet to commence antihypertensives or had been taking antihypertensive medications for less than 6-month duration were excluded from this study. Each respondent's sexual functions were assessed using a structured interviewer administered questionnaire validated by the International Index of Erectile Function (IIEF). All the data collected were analyzed using the Statistical Package for Social Sciences (SPSS) version 17 (released 2008; SPSS Incorporations, IL, USA). Results were presented in tables. Discrete variables were presented as frequency and percentages. Continuous variables were presented as mean ± standard deviation. Proportions were calculated for each categorical variable and associations between those categorical variables were tested using chi-square. For all tests, p-value < 0.05 was considered statistically significant.

Ethical clearance was obtained from LUTH Health Research Ethical Committee before commencing this study. In addition, a duly signed written informed consent was obtained from each of the patients whose medical records were used, while the medical records for those who did not sign their informed consent were excluded from the study. Participants' confidentiality was respected and maintained by ensuring that no unauthorized person had access to the information on the data information sheets, that no information can be traced to the subjects (as coding system was used for the data information sheets instead of writing the patients' names on them) and no unauthorized use of information was made.

## Results

Table 1 shows the socio-demographic data for the respondents. The respondents were between the age range of 30 and 98 years with a mean of 59 ± 11.1 years (modal of 51 years). Their weight distribution was between the range of 51 and 110 kg with a mean of 72 ± 14.2 kg (modal of 61 kg). Regarding the respondent's blood pressure during their initial medical diagnosis for hypertension; their systolic blood pressure (SBP) was between the range of 128 and 194 mmHg with a mean of 162 ± 16.4 mmHg (modal of 160 mmHg). While their diastolic blood pressure (DBP) was between the range of 78 and 120 mmHg with a mean of 95 ± 10.7 mmHg (modal of 90 mmHg).

**Table 1.** Socio-demographic and clinical characteristics of the respondents.

Characteristics	Mean	SD	Min	Max	Mode
Age (years)	59	11.1	30	98	51
Weight (kg)	72	14.2	51	110	61
Systolic BP (mmHg)	162	16.4	128	194	160
Diastolic BP (mmHg)	95	10.7	78	120	90

Min: Minimum; Max: Maximum; SD: Standard deviation.

Table 2 shows the frequency distribution pattern for the antihypertensive medications used by the respondents. The most frequently prescribed antihypertensive medications were calcium channel blockers (CCBs) in 68 (42.8%) patients, followed by diuretics in 66 (41.5%) patients, angiotensin converting enzyme inhibitors (ACE inhibitors) in 50 (31.4%) patients, angiotensin II receptor antagonists in 44 (27.7%) patients, beta receptor blockers in 17 (10.7%) patients and centrally acting antihypertensives in 17 (10.7%) patients. The rest of other prescribed antihypertensive medications are shown in Table 2.

**Table 2.** Frequency distribution pattern for the antihypertensive medications used by the respondents.

Classes	Frequency (%)
Calcium channel blockers	68 (42.8)
Diuretics	66 (41.5)
Angiotensin converting enzyme inhibitors	50 (31.4)
Angiotensin II receptor antagonists	44 (27.7)
Beta receptor blockers	17 (10.7)
Centrally acting antihypertensives	17 (10.7)
Combination of beta receptor blocker and diuretic (such as; atenolol and chlorthalidone [tenoric])	3 (1.9)
Combined alpha- and beta-receptor blocker antihypertensives	2 (1.3)
Vasodilators	2 (1.3)
Alpha receptor blockers	1 (0.6)

Table 3 illustrates types and severity of sexual dysfunctions among the respondents.

**Table 3.** Types and severity of male sexual dysfunctions.

Degree of severity for male sexual dysfunctions	Male sexual dysfunctions		
	Erectile dysfunction (%)	Intercourse dissatisfaction (%)	Orgasmic dysfunction (%)
Severe	10 (6.3)	19 (11.9)	13 (8.2)
Moderate	9 (5.7)	24 (15.1)	34 (21.4)
Mild-to-moderate	62 (39.0)	47 (29.6)	30 (18.9)
Mild	58 (36.5)	56 (35.2)	56 (35.2)
None	20 (12.6)	13 (8.2)	26 (16.4)
Total	159 (100.0)	159 (100.0)	159 (100.0)

Regarding erectile function among the participants, there was mild-to-moderate erectile dysfunction in 62 (39.0%) patients, followed by mild erectile dysfunction in 58 (36.5%) patients, severe erectile dysfunction in 10 (6.3%) patients, moderate erectile dysfunction in 9 (5.7%) patients, while 20 (12.6%) patients had adequate erectile function.

Concerning intercourse satisfaction among the participants, there was mild intercourse dissatisfaction in 56 (35.2%) patients, followed by mild-to-moderate intercourse dissatisfaction in 47 (29.6%) patients, moderate intercourse dissatisfaction in 24 (15.1%) patients, severe intercourse dissatisfaction in 19 (11.9%) patients, while 13 (8.2%) patients had adequate intercourse satisfaction.

Considering orgasmic function among the participants, there was mild orgasmic dysfunction in 56 (35.2%) patients, followed by moderate orgasmic dysfunction in 34 (21.4%) patients, mild-to-moderate orgasmic dysfunction in 30 (18.9%) patients, severe orgasmic dysfunction in 13 (8.2%) patients, while 26 (16.4%) patients had adequate orgasmic function.

Table 4 compared the pattern of usage of sexual stimulating drugs for male sexual dysfunctions (erectile dysfunction, intercourse dissatisfaction and orgasmic dysfunction) and severity of these dysfunctions among the respondents.

**Table 4.** Comparing usage pattern of sexually stimulating drugs and severity of male sexual dysfunctions among the respondents.

Degree of severity for male sexual dysfunctions	Male sexual dysfunctions								
	Erectile dysfunction			Intercourse dissatisfaction			Orgasmic dysfunction		
	Sexual stimulating drug(s) used (%)	Sexual stimulating drug(s) not used (%)	Total number of patients with erectile dysfunction	Sexual stimulating drug(s) used (%)	Sexual stimulating drug(s) not used (%)	Total number of patients with intercourse dissatisfaction	Sexual stimulating drug(s) used (%)	Sexual stimulating drug(s) not used (%)	Total number of patients with orgasmic dysfunction
Severe	0 (0.0)	10 (6.3)	10 (6.3)	1 (0.6)	18 (11.3)	19 (11.9)	0 (0.0)	13 (8.2)	13 (8.2)
Moderate	5 (3.1)	4 (2.5)	9 (5.7)	8 (5.0)	16 (10.1)	24 (15.1)	10 (6.3)	24 (15.1)	34 (21.4)
Mild-to-moderate	22 (13.8)	40 (25.2)	62 (39.0)	20 (12.6)	27 (17.0)	47 (29.6)	14 (8.8)	16 (10.1)	30 (18.9)
Mild	25 (15.7)	33 (20.8)	58 (36.5)	27 (17.0)	29 (18.2)	56 (35.2)	30 (18.9)	26 (16.4)	56 (35.2)
None	8 (5.0)	12 (7.5)	20 (12.6)	4 (2.5)	9 (5.7)	13 (8.2)	7 (4.4)	19 (12.0)	26 (16.4)

None of the 10 (6.3%) patients with severe erectile dysfunction made use of sexual stimulating drug(s). Out of the 9 (5.7%), 62 (39.0%) and 58 (36.5%) patients with moderate, mild-moderate and mild erectile dysfunction, respectively, 5 (3.1%), 22 (13.8%) and 25 (15.7%) made use of sexual stimulating drug(s), while 4 (2.5%), 40 (25.2%) and 33 (20.8%) did not. Out of the 20 (12.6%) patients with adequate erectile function, 8 (5.0%) made use of sexual stimulating drug(s), while the remaining 12 (7.5%) did not make use of any sexual stimulating drug (Table 4).

Out of 19 (11.9%), 24 (15.1%), 47 (29.6%) and 56 (35.2%) patients with severe, moderate, mild-to-moderate, mild intercourse dissatisfaction respectively, 1 (0.6%), 8 (5.0%), 20 (12.6%) and 27 (17.0%) made use of sexual stimulating drug(s), while 18 (11.3%), 16 (10.1%), 27 (17.0%) and 29 (18.2%) did not make use of any sexual stimulating drug. Out of the 13 (8.2%) patients with adequate intercourse satisfaction, 4 (2.5%) made use of sexual stimulating drug(s), while the remaining 9 (5.7%) did not make use of any sexual stimulating drug (Table 4).

None of the 13 (8.2%) patients with severe orgasmic dysfunction made use of sexual stimulating drug(s). Also, among the 34, 30 and 56 patients representing 21.4, 18.9 and 35.2% respectively of these respondents with moderate, mild-to-moderate and mild orgasmic dysfunction; those that made use of sexual stimulating drug(s) were 10 (6.3%), 14 (8.8%) and 30 (18.9%), while 24 (15.1%), 16 (10.1%) and 26 (16.4%) did not make use of any sexual stimulating drug. Out of the 26 (16.4%) patients with adequate orgasmic function, 7 (4.4%) patients used sexual stimulating drug(s) while the remaining 19 (12.0%) patients did not make use of any sexual stimulating drug (Table 4).

Table 5 illustrates the sexual desire status during the intake of different antihypertensive medications. The highest occurrence of male sexual dysfunctions was produced by calcium channel blockers (CCBs) in 32 (20.1%) patients, followed by diuretics in 27 (17.0%) patients, ACE Inhibitors in 20 (12.6%) patients, centrally acting antihypertensives in 11 (6.9%) patients, beta receptor blockers in 8 (5.0%) patients, vasodilators in 2 (1.3%) patients, alpha- and beta-receptor blockers antihypertensives in 2 (1.3%) patients and alpha receptor blockers in 1 (0.6%) patients.

**Table 5.** Cross tabulation of sexual desire (activity) status during the intake of different antihypertensive medications.

Desire for sexual activity	ACE inhibitors (%)	Alpha receptor blockers (%)	Beta receptor blockers (%)	Calcium channel blockers (%)	Centrally acting antihypertensives (%)	Diuretics (%)	Vasodilators (%)	Combined alpha- and beta-receptor blockers antihypertensives (%)
No change in desire	30 (18.9)	0 (0.0)	9 (5.7)	36 (22.6)	6 (3.8)	38 (23.9)	0 (0.0)	0 (0.0)
Decrease in desire	20 (12.6)	1 (0.6)	8 (5.0)	32 (20.1)	11 (6.9)	27 (17.0)	2 (1.3)	2 (1.3)

ACE: Angiotensin converting enzyme inhibitor.

Table 6A revealed that there was a statistically significant association between sexual desire status and erectile function among the respondents with a p-value of 0.01903.

**Table 6A.** Sexual desire status and erectile function.

Decrease in desire for sexual activity	Severe	Moderate	Mild-to-moderate	Mild	No dysfunction
No change in desire	4	1	30	36	14
Decrease in desire	6	8	32	22	6

Chi-X^2^ = 11.78; df = 4; p = 0.01903 (significant); critical value = 9.488; α = 0.05.

Furthermore, Table 6B revealed that there was a statistically significant association between sexual desire status and intercourse satisfaction among the respondents with a p-value of 0.01802.

**Table 6B.** Sexual desire status and intercourse satisfaction.

Decrease in desire for sexual activity	Severe	Moderate	Mild-to-moderate	Mild	No dysfunction
No change in desire	11	9	18	38	8
Decrease in desire	8	15	29	18	5

Chi-X^2^ = 11.91; df = 4; p = 0.01802 (significant); critical value = 9.488; α = 0.05.

In addition, Table 6C revealed that there was a statistically significant association between sexual desire status and orgasmic function among the respondents with a p-value of 0.003549.

**Table 6C.** Sexual desire status and orgasmic function.

Decrease in desire for sexual activity	Severe	Moderate	Mild-to-moderate	Mild	No dysfunction
No change in desire	5	19	9	30	21
Decrease in desire	8	15	21	26	5

Chi-X^2^ = 15.64; df = 4; p = 0.003549 (significant); critical value = 9.488; α = 0.05.

Also, Table 6D revealed that there was a statistically significant association between the degree of severity for intercourse dissatisfaction and the use of sexual stimulating drug(s) status among the respondents with a p-value of 0.01680.

**Table 6D.** Degree of severity for intercourse dissatisfaction and use of sexual stimulating drug(s) status.

Degree of severity for intercourse dissatisfaction	Sexual stimulating drug(s) used (%)	Sexual stimulating drug(s) not used (%)
Severe	1	18
Moderate	8	16
Mild-to-moderate	20	27
Mild	27	29
None	4	9

Chi-X^2^ = 12.07; df = 4; p = 0.01680 (significant); critical value = 9.488; α = 0.05.

## Discussion

This study was designed to assess the effect of antihypertensive medications use on the preservation of adequate sexual function among hypertensive adult male patients attending the medical outpatient clinic of LUTH, Nigeria. The results from this study revealed that calcium channel blockers (CCBs) were the most frequently administered antihypertensive medications in 68 (42.8%) patients, followed by diuretics in 66 (41.5%) patients, ACE inhibitors in 50 (31.4%) patients, angiotensin II receptor antagonists in 44 (27.7%) patients, beta receptor blockers in 17 (10.7%) patients and centrally acting antihypertensives in 17 (10.7%) patients. Despite the fact that CCBs was the most frequently prescribed antihypertensive medications in this study, it was also observed that majority of the respondents who experienced sexual dysfunctions were on a CCB-containing antihypertensive medication combinations. Furthermore, it was observed that the use of calcium channel blockers CCBs was associated with the highest occurrence of sexual dysfunctions, followed by diuretics, ACE inhibitors and centrally acting antihypertensives.

The mechanism of action for CCBs is that they inhibit the influx of calcium ions into the heart myocardium, this occurs with the use of verapamil and diltiazem [2,3,7–9], or/and arteriolar smooth muscles as in the case of nifedipine, amlodipine, nimodipine, felodipine and diltiazem [2,3,10–12]. They act by suppressing myocardial contractility (verapamil and diltiazem) to result in a reduced heart rate, reduced cardiac output [2,3,13–19] or by dilating the blood arterioles to result in a reduced effective total peripheral vascular resistance and tone of the arterioles with CCBs such as the dihydropyridines (nifedipine, amlodipine, nimodipine, felodipine) and diltiazem [2,3,16–23]. This makes it easier for the heart to pump blood with less stress through less resistant arterioles. Diuretics were second to CCBs concerning the tendency of association with sexual dysfunctions, while ACE inhibitors [14,15] were the next most frequent class of antihypertensive medications associated with sexual dysfunctions. Moreover, gynecomastia and problem with ejaculation had been reported with a few diuretic therapy such as spironolactone [1–3,5,6].

Several widely prescribed antihypertensive agents, including diuretics, methyldopa, clonidine, guanethidine and beta receptor blockers (especially those that are nonselective), are known to cause sexual problems or exacerbate existing sexual dysfunctions [25–30]. However, not all classes of antihypertensive agents share the same risk of inducing sexual dysfunctions [31–34], and certain classes of antihypertensive agents tend to be associated with a higher prevalence of sexual dysfunctions than others [35–38]. Differences among the various classes of antihypertensive agents have been noted in men with respect to erectile dysfunction, decreased libido, intercourse dissatisfaction and impairment of ejaculation [39–44]. This is one of the factors responsible for the lack of recognition of sexual dysfunctions as a component of the hypertensive process rather than as a consequence of antihypertensive medications [45–48]. Compared with placebo or other classes of antihypertensive agents, a higher prevalence of male sexual dysfunctions have been reported in some studies of diuretics, including spironolactone, which inhibits dihydrotestosterone binding, and thiazide diuretics (e.g., chlorthalidone), as well as beta receptor blockers [49–51]. Centrally acting antihypertensives such as methyldopa and clonidine may potentially impact sexual functioning through a variety of mechanisms, including a decrease in central sympathetic outflow, impairment of vasodilation of the corpora cavernosa, effects on luteinizing hormone and testosterone secretion and a tendency to produce sedation and depression, thereby causing loss of libido and ejaculation impairment [50–53]. However, as noted, deleterious effects of diuretics and beta receptor blockers on sexual functions have not been consistently established, and several controlled studies, including a combined analysis of six randomized, blinded, prospective trials, have found little or no evidence for a greater risk of occurrence of adverse sexual dysfunctions between these agents and other antihypertensive medications [50–54]. Moreover, only CCBs, diuretics and ACE inhibitors were found to be prescribed more in LUTH compared with these previous studies [1–5,54].

The socio-demographic findings from this study also demonstrate that the average age for hypertensive male patients was approximately 59 years, and the average weight of these men was about 72 kg. We can deduce from this observation that increasing weight (which results in increasing body mass index) and aging process are predisposing risk factors for the occurrence of hypertension among adult males, as revealed in previous studies done in other parts of the world [1,4,7–10,21].

This study revealed that there was a statistically significant association between sexual desire status and erectile function among the respondents with a p-value of 0.01903. Furthermore, there was a statistically significant association between sexual desire status and intercourse satisfaction among the respondents with a p-value of 0.01802. In addition, there was a statistically significant association between sexual desire status and orgasmic function among the respondents with a p-value of 0.003549. Also, there was a statistically significant association between the degree of severity for male intercourse dissatisfactions and the use of sexual stimulating drug(s) among the respondents with a p-value of 0.01680.

The limitation and strength of this study was that it only considered patients who gave their consent and were hypertensive adult male medical patients on antihypertensive medications for at least 6-month duration; while newly diagnosed hypertensive adult male medical patients that were yet to commence antihypertensive medications or had been taking antihypertensive medications for less than 6-month duration were completely excluded from this study. In addition, hypertensive adult male medical patients who declined to be enrolled in the study, hypertensive adult male medical patients with cognitive impairment, non-hypertensive adult male medical patients, pediatric unit patients, who are less than 18 years old and surgical unit patients were completely excluded from this study. Finally, all the observed results are completely and exclusively applicable to only hypertensive adult male medical patients in clinical practice setting.

## Conclusion

In this study, it was found that CCBs use was associated with the highest occurrence of sexual dysfunctions, followed by diuretics, ACE inhibitors and centrally acting antihypertensives. Furthermore, the most frequent antihypertensive drugs prescribed for hypertensive adult male medical patients in this study was found to be CCBs, followed by diuretics, ACE inhibitors and angiotensin II receptor antagonists. We advocate that medical practitioners should consider choosing antihypertensive therapy with the lowest possible potential for adverse drug effects such as sexual dysfunctions among adult hypertensive men in order to attain an optimum balance between antihypertensive efficacy and adequate preservation of sexual functions. Finally, for all persons with hypertension, the potential benefits of a healthy diet, weight control and regular exercise cannot be overemphasized. These lifestyle modifications have the potential to improve blood pressure control and even reduce medication needs. This reduces adverse drug effects in these patients.

## Future perspective

Though, the mechanisms by which antihypertensive medications control patients' blood pressure are known, the same basis may not necessarily apply for example as regards the blood vessels for erectile function, hence, it is necessary to establish the exact mechanisms of these medications in sexual dysfunctions. Such knowledge will be useful in classification of mechanisms of action of different antihypertensives as regards sexual dysfunctions, types of sexual dysfunction(s) expected, which medications may not be prescribed concomitantly and other probable options to counteract the manifestation of sexual dysfunction(s).

**Summary points**

- Hypertension, which is one of the most prevalent diseases in elderly patients (often 60 years and above), has been reported to affect 8–46.4% of the Nigerian population according to the *World Journal of Cardiology*. The management of hypertensive patients should take into account especially the impact of antihypertensive drugs on patients' sexual functions, the deterioration of which may result in reduced adherence to treatment. This study was designed to assess the effect of prescribed antihypertensive medications on sexual functions among hypertensive adult male patients attending the medical outpatient clinic of Lagos University Teaching Hospital, Nigerian.
- This was a descriptive, prospective study among 159 hypertensive adult male patients who had been on antihypertensive medications for at least 6-month duration and agreed to enroll.
- The highest occurrence of sexual dysfunctions was associated with calcium channel blockers in 32 (20.1%) patients, followed by diuretics in 27 (17.0%) patients, angiotensin converting enzyme inhibitors in 20 (12.6%) patients, centrally acting antihypertensives in 11 (6.9%) patients, beta receptor blockers in 8 (5.0%) patients, vasodilators in 2 (1.3%) patients, combined alpha- and beta-receptor blocker antihypertensives in 2 (1.3%) patients and alpha receptor blockers in 1 (0.6%) patients.
- Medical practitioners should consider choosing antihypertensive medications with the lowest possible potential for adverse drug effects such as sexual dysfunctions among adult hypertensive men in order to attain an optimum balance between antihypertensive efficacy and adequate preservation of sexual functions. The foregoing combined with lifestyle modifications have the potential to improve blood pressure control and even reduce medication needs, ultimately, adverse drug effects among these patients will be reduced.

## References

[1] Tanner LA, Bosco LA Gynecomastia associated with calcium channel blocker therapy. Arch. Intern. Med. 148, 379–380 (1988). 3341839

[2] Fogari R, Zoppi A, Corradi L Sexual function in hypertensive males treated with lisinopril or atenolol: a cross-over study. Am. J. Hypertens. 11(10), 1244–1247 (1998). 9799042 10.1016/s0895-7061(98)00139-3

[3] Benedict CR Safe and effective management of hypertension with fixed-dose combination therapy: focus on losartan plus hydrochlorothiazide. Intl. J. Clin. Pract. 54, 48–54 (2000). 10750261

[4] Moore MA, Edelman JM, Gazdick LP Choice of initial antihypertensive medication may influence the extent to which patients stay on therapy: a community-based study of the tolerability and effectiveness of a losartan-based regimen versus usual care. High Blood Press. 7, 156–157 (1998).

[5] Bansal S Sexual dysfunction in hypertensive men: a critical review of the literature. Hypertension 12, 1–10 (1998). 10.1161/01.hyp.12.1.1 3294176

[6] Benet AE, Melman A The epidemiology of erectile dysfunction. Urol. Clin. North Am. 22, 699–709 (1995). 7483123

[7] Billups KL Endothelial dysfunction as a common link between erectile dysfunction and cardiovascular disease. Current Sexual Health Reports 1, 137–141 (2004).

[8] Blumentals WA, Gomez-Caminero A, Joo S Should erectile dysfunction be considered as a marker for acute myocardial infarction? Results from a retrospective cohort study. Int. J. Impot. Res 16, 350–353 (2004). 14985780 10.1038/sj.ijir.3901174

[9] Bohlen JG, Held JP, Sanderson MO Heart rate, rate-pressure product and oxygen uptake during four sexual activities. Arch. Intern. Med. 14, 1745–1748 (1984). 6476990

[10] Brock H, McMahon CG, Chen KK Efficacy and safety of tadalafil for the treatment of erectile dysfunction: results of integrated analyses. J. Urol. 168, 1332–1336 (2002). 12352386 10.1016/S0022-5347(05)64442-4

[11] Bruckert E, Giral P, Heshmati HM Men treated with hypolipidaemic drugs complain more frequently of erectile dysfunction. J. Clin. Pharm. Ther. 21, 89–94 (1996). 8809645 10.1111/j.1365-2710.1996.tb00006.x

[12] Carvajal A, Lerida MT, Sanchez A ACE inhibitors and impotence: a case series from Spanish drug monitoring system. Drug Saf. 13, 130–131 (1995). 7576264 10.2165/00002018-199513020-00007

[13] Cardillo C, Kilcoyne CM, Quyyumi AA A selective defect in nitric oxide synthesis may explain the impaired endothelium dependent vasodilation in patients with essential hypertension. Circulation 97, 851–856 (1998). 9521333 10.1161/01.cir.97.9.851

[14] Cheitlin MD, Hutter AM, Brindis RG ACC/AHA Expert consensus document. Use of sildenafil (Viagra) in patients with cardiovascular disease. J. Am. Coll. Cardiol. 33, 273–282 (1998). 10.1016/s0735-1097(98)00656-1 9935041

[15] DeBusk RF Sexual activity triggering myocardial infarction: one less thing to worry about. JAMA 275, 1447–1448 (1996). 8618373

[16] DeBusk R, Drory Y, Goldstein I Management of sexual dysfunction in patients with cardiovascular disease: recommendations of the Princeton Consensus Panel. Am. J. Cardiol. 86, 175–181 (2000). 10913479 10.1016/s0002-9149(00)00896-1

[17] DeBusk RF Evaluating the cardiovascular tolerance to sex. AmJ Cardiol 86(Suppl.), 51F–56F (2000). 10.1016/s0002-9149(00)00894-8 10899280

[18] Derby CA, Mohr BA, Goldstein I Modifiable risk factors and erectile dysfunction: can lifestyle changes modify risk? Urology 56, 302–306 (2000). 10925098 10.1016/s0090-4295(00)00614-2

[19] Moulik PK, Hardy KJ Hypertension, anti-hypertensive drug therapy and erectile dysfunction in diabetes. Diabet. Med. 20(4), 290–293 (2003). 12675642 10.1046/j.1464-5491.2003.00911.x

[20] Hale TM, Okabe H, Heaton JP Antihypertensive drugs induce structural remodeling of penile vasculature. J. Urol. 166, 739–745 (2001). 11458127

[21] Dusing R Effect of the angiotensin II antagonist valsartan on sexual function in hypertensive men. Blood Press Suppl 2, 29–34 (2003). 14761074 10.1080/08038020310021967

[22] Feldman HA, Goldstein I, Hatzichristou DG Impotence and its medical and psychosocial correlates: results of the Massachusetts Male Aging Study. J. Urol. 151, 54–61 (1994). 8254833 10.1016/s0022-5347(17)34871-1

[23] Ferrario CM, Levy P Sexual function in patients with hypertension: implications for therapy. J. Clin. Hypertens 4, 424–432 (2002). 10.1111/j.1524-6175.2002.00862.x PMC8101845 12461307

[24] Jackson G Sexual intercourse and stable angina pectoris. Am. J. Cardiol. 86(Suppl.), 35F–37F (2000). 10899276 10.1016/s0002-9149(00)00890-0

[25] Johannes CB, Araujo AB, Feldman HA Incidence of erectile dysfunction in men 40 to 69 years old: longitudinal results from the Massachusetts Male Aging Study. J. Urol. 163, 460–463 (2000). 10647654

[26] Kaiser DR, Billups K, Mason C Impaired brachial artery endothelium-dependent and -independent vasodilation in men with erectile dysfunction and no other clinical cardiovascular disease. J. Am. Coll. Cardiol. 43, 179–184 (2004). 14736434 10.1016/j.jacc.2003.07.042

[27] Keene LC, Davies PH Drug-related erectile dysfunction. Adverse Drug React. Toxicol. Rev. 18, 5–24 (1999). 10401520

[28] Kim SW, Paick J, Park DW Potential predictors of asymptomatic ischemic heart disease in patients with vasculogenic erectile dysfunction. Urology 58, 441–445 (2001). 11549496 10.1016/s0090-4295(01)01210-9

[29] Kloner RA, Jarow JP Erectile dysfunction and sildenafil citrate and cardiologists. Am. J. Cardiol. 83, 576–582 (1999). 10073864 10.1016/s0002-9149(98)00916-3

[30] Kloner RA Hypertension as a risk for erectile dysfunction: implications for sildenafil use. J. Clin. Hypertens 2, 33–36 (2000). 11416623

[31] Toblli JE, Stella I, Inserra F Morphological changes in cavernous tissue in spontaneously hypertensive rats. Am. J. Hypertens. 13(6 pt 1), 686–692 (2000). 10912754 10.1016/s0895-7061(99)00268-x

[32] Burchardt M, Burchardt T, Baer L Hypertension is associated with severe erectile dysfunction. J. Urol. 164(4), 1188–1191 (2000). 10992363

[33] Prisant LM, Weir MR, Frishman WH Self reported sexual dysfunction in men and women treated with bisoprolol, hydrochlorothiazide, enalapril, amlodipine, placebo, or bisoprolol/hydrochlorothiazide. J. Clin. Hypertens. 1(1), 22–26 (1999). 11416589

[34] Lewis C, Duncan LE, Ballance DI Is sexual dysfunction in hypertensive women uncommon or understudied? Am. J. Hypertens. 11(6 pt 1), 733–735 (1998). 9657635

[35] Medical Research Council Working Party. MRC trial of treatment of mild hypertension: principal results. Br. Med. J. 29, 97–104 (1985). 10.1136/bmj.291.6488.97 PMC1416260 2861880

[36] Williams GH Quality of life and its impact on hypertensive patients. Am. J. Med. 82(1), 98–105 (1987). 3799696 10.1016/0002-9343(87)90382-2

[37] Jachuck SJ, Brierley H, Jachuck S The effect of hypotensive drugs on the quality of life. J. R. Coll. Gen. Pract. 32, 103–105 (1982). 7097628 PMC1971011

[38] Wassertheil-Smoller S, Blaufox MD, Oberman A Effect of antihypertensives on sexual function and quality of life: the TAIM Study. Ann. Intern. Med. 114(8), 613–620 (1991). 2003706 10.7326/0003-4819-114-8-613

[39] Bansal S Sexual dysfunction in hypertensive men. A critical review of the literature. Hypertension 12, 1–10 (1988). 3294176 10.1161/01.hyp.12.1.1

[40] Lue TF Erectile dysfunction. N. Engl. J. Med. 342, 1802–1813 (2000). 10853004 10.1056/NEJM200006153422407

[41] Suzuki H, Tominaga T, Kumagai H Effects of first-line antihypertensive agents on sexual function and sex hormones. J. Hypertens. Suppl. 6(4), S649–S651 (1988). 3149291 10.1097/00004872-198812040-00204

[42] Muller SC, el-Damanhoury H, Ruth J Hypertension and impotence. Eur. Urol. 19(1), 29–34 (1991). 2007414 10.1159/000473574

[43] Kroner BA, Mulligan T, Briggs GC Effect of frequently prescribed cardiovascular medications on sexual function: a pilot study. Ann. Pharmacotherapy. 27, 1329 (1993). 10.1177/106002809302701103 8286802

[44] Neaton JD, Grimm RH Jr, Prineas RJ Treatment of mild hypertension study. JAMA 270, 713–724 (1995). 8336373

[45] Rosen RC, Kostis JB, Jekelis A Sexual sequelae of antihypertensive drugs: treatment effects on self-report and physiological measures in middle-aged male hypertensives. Arch. Sex. Behav. 23(2), 135–152 (1994). 8018020 10.1007/BF01542095

[46] Bloom BS Continuation of initial antihypertensive medication after 1 year of therapy. Clin. Ther. 20, 671–681 (1998). 9737827 10.1016/s0149-2918(98)80130-6

[47] Tedesco MA, Ratti G, Mennella S Comparison of losartan and hydrochlorothiazide on cognitive function and quality of life in hypertensive patients. Am. J. Hypertens. 12(11 pt 1), 1130–1134 (1999). 10604491 10.1016/s0895-7061(99)00156-9

[48] Bulpitt CJ, Dollery CT Side effects of hypotensive agents evaluated by a self-administered questionnaire. Br. Med. J. 3, 485–490 (1973). 4726158 10.1136/bmj.3.5878.485 PMC1586624

[49] Hogan MJ, Wallin JD, Baer RM Antihypertensive therapy and male sexual dysfunction. Psychosomatics 21, 234–237 (1980). 7189289 10.1016/S0033-3182(80)73698-8

[50] Curb JD, Borhani NO, Blaszkowski TP Long-term surveillance for adverse effects of antihypertensive drugs. JAMA 253, 3263–3268 (1985). 3999311

[51] Scharf MB, Mayleben DW Comparative effects of prazosin and hydrochlorothiazide on sexual function in hypertensive men. Am. J. Med. 86(1B), 110–112 (1989). 10.1016/0002-9343(89)90144-7 2913766

[52] Chang SW, Fine R, Siegel D The impact of diuretic therapy on reported sexual function. Arch. Intern. Med. 151(12), 2402–2408 (1991). 1746997

[53] Kochar MS, Mazur LI, Patel A What is causing your patients sexual dysfunction? Uncovering a connection with hypertension and antihypertensive therapy. Postgrad Med. 106(2), 149–157 (1999). 10.3810/pgm.1999.08.655 10456046

[54] Fogelman J Verapamil caused depression, confusion, and impotence. Am. J. Psychiatry 145(3), 380 (1988). 10.1176/ajp.145.3.380b 3344858

